# Psychoactive Plant Database: a phytochemical resource for neurological drug discovery

**DOI:** 10.3389/fphar.2025.1569127

**Published:** 2025-05-23

**Authors:** Harjot Kaur, Monika Gupta, Zabeer Ahmed, Amit Nargotra

**Affiliations:** ^1^ Discovery Informatics, NPMC Division, CSIR-Indian Institute of Integrative Medicine, Jammu, India; ^2^ Academy of Scientific and Innovative Research (AcSIR), Ghaziabad, India; ^3^ Pharmacology Division, CSIR-Indian Institute of Integrative Medicine, Jammu, India

**Keywords:** psychoactive plants, neurodegenerative diseases, compound properties, traditional knowledge digital library, psychoactive plant database, nucleotide-binding domain, leucine-rich repeat-containing family, pyrin domain-containing-3

## 1 Introduction

Psychoactive plants are rich sources of bioactive compounds that modulate central nervous system (CNS) activity and have shown therapeutic potential for neurological and psychiatric disorders, including Alzheimer’s disease (AD), Parkinson’s disease (PD), epilepsy, and depression. These disorders pose significant global health challenges, necessitating the development of innovative therapeutic strategies (Puri et al., 2022; Feigin et al., 2020). Various plant families, such as Solanaceae (*Atropa belladonna* L. and *Datura stramonium* L.), Papaveraceae (*Papaver somniferum* L.), and Fabaceae (*Mimosa pudica* L.), produce neuroactive phytochemicals with well-documented CNS effects (Moise et al., 2024; Gutiérrez-Del-Río et al., 2021). Several classes of phytochemicals have been identified as potential therapeutic agents for neurological disorders. Alkaloids such as scopolamine and hyoscyamine exert anticholinergic properties (Kohnen-Johannsen et al., 2019), while morphine, an opiate alkaloid, remains a potent analgesic (Khademi et al., 2016). Phenolic compounds, including quercetin and curcumin, demonstrate neuroprotective properties by attenuating oxidative stress and neuroinflammation (Rojas-García et al., 2023). Additionally, terpenoids, such as cannabidiol (CBD) from *Cannabis sativa* L., exhibit anxiolytic, anti-inflammatory, and neuroprotective effects, modulating key neurotransmitter pathways such as dopaminergic and serotonergic signaling (Stasiłowicz-Krzemień et al., 2024; Rehman et al., 2019).

The significance of plant-derived compounds in drug discovery is well-established, with approximately 25% of clinically approved drugs originating from natural products. Notably, natural compounds contribute to 60% of anticancer agents and 70% of anti-infective drugs, highlighting their continued relevance in modern pharmacology (Nasim et al., 2022; Asma et al., 2022;Newman et al., 2020). Moreover, over 30% of pharmaceuticals currently in clinical trials are derived from natural sources, emphasizing the role of plant-based bioactive compounds in advancing novel therapeutic interventions.

Despite the therapeutic potential of psychoactive plant-derived compounds for neurological and neurodegenerative disorders, existing knowledge on these phytochemicals remains fragmented and dispersed across multiple sources. This lack of a centralized, disease-specific database poses significant challenges in drug discovery and mechanistic research (Domingo-Fernandez D. et al., 2023; Domingo-Fernández D. et al., 2024; Kim Y. C., 2010; Andrade S. et al., 2023). There are a few phytochemical databases such as the Dictionary of Natural Products (DNP) (Natural Product, 2015) [http://dnp.chemnetbase.com], KNApSAcK (Afendi et al., 2012), Dr. Duke’s Phytochemical and Ethnobotanical Databases (U.S. Department of Agriculture, Argicultural Research Service, 2013) [http://phytochem.nal.usda.gov], and IMPPAT (Vivek-Ananth et al., 2023), but most of these provide data on plant-derived compounds, in general, and lack disease-specific insights. Other databases, such as CANNUSE (Balant et al., 2021), are focused only on cannabis-related compounds, while EROWID (Erowid, 2020) [Erowid.org] provides generalized data on only the psychoactive substance (Gonçalves et al., 2021; Ryan, 2020; Spinks et al., 2011; Khadka et al., 2020).

The Psychoactive Plant Database (PPD), a specialized platform focused on all the known phytochemicals isolated from psychoactive plants with a specific emphasis on their role in neurological disorders, has been developed to address these limitations. Unlike existing resources, PPD systematically compiles taxonomically validated species (*MPNS Kew*, *POWO*) (POWO, 2025) [https://mpns.science.kew.org; https://powo.science.kew.org]; curated phytochemical data relevant to CNS activity; binding affinity of all the phytochemicals against NLRP3, a critical protein in neuroinflammation; comprehensive literature references from peer-reviewed studies; *in silico* ADMET predictions, aiding in pharmacokinetic and drug-likeness assessments; advanced search features, enabling retrieval of phytochemicals based on plant species, molecular weight, CAS ID, chemical class, and biological activity. In addition to these features, the database also correlates the phytochemicals with the existing drugs, which can be very useful information for drug repurposing.

PPD is the first disease-centric database that integrates ethnobotanical knowledge with computational pharmacology, providing a scientifically rigorous and disease-relevant platform for researchers. By consolidating fragmented information into a single, structured resource, PPD facilitates interdisciplinary collaboration that can accelerate the discovery of novel neuroprotective agents for neurological and neurodegenerative disorders.

## 2 Database overview

The Psychoactive Plant Database is a comprehensive, unified platform to consolidate information on psychoactive plants and their phytochemicals, facilitating neurodegenerative drug discovery. PPD compiles extensive data on 124 therapeutic medicinally relevant psychoactive plants from 60 plant families, encompassing over 7,000 phytochemicals. To ensure accuracy and reliability, all phytochemical data were meticulously collected from four authenticated databases: IMPPAT, Dr. Duke’s Phytochemical and Ethnobotanical Databases, KNApSAcK, and the Dictionary of Natural Products (DNP). Duplicate phytochemical entries from each plant were carefully removed, and each phytochemical was cross-referenced with its respective data source and supporting scientific literature. The scientific plant names were validated using World Flora Online (WFO) (WFO, 2023) and cross-verified through additional botanical databases such as MPNS [https://mpns.science.kew.org] and POWO (POWO, 2025) [https://powo.science.kew.org]. Among the 124 selected psychoactive plants, 31 species—including *Piper longum* L. and *Withania somnifera* (L.) Dunal—have been historically used in formulations targeting nervous system disorders. Notably, the Solanaceae family stands out with 14 psychoactive plants enriched with tropane alkaloids such as scopolamine and hyoscyamine, which are well known for their neurological applications (Jaremicz Z. et al., 2014).

To enhance the therapeutic relevance of the database, *in silico* absorption, distribution, metabolism, excretion, and toxicity (ADMET) profiling was carried out, with a particular focus on CNS activity. It was found that 29% of the phytochemicals exhibited predicted CNS-active properties with favorable pharmacokinetics, including molecular weights below 500 g/mol, making them promising candidates for further neurotherapeutic exploration. Furthermore, molecular docking studies were performed against NLRP3, a key target in neuroinflammation and neurodegenerative diseases, to determine the binding affinity of all the phytochemicals against this important neuroinflammation target. Several compounds demonstrated strong binding affinity (low docking scores), highlighting their potential as neuroprotective agents. Docking analysis plays a crucial role in predicting molecular interactions, allowing for the identification of lead compounds with high target specificity and binding strength. These computational insights help prioritize phytochemicals for *in vitro* and *in vivo* validation, accelerating their potential translation into therapeutic agents for neurological disorders. The database also integrates structural and pharmacological information for each collected phytochemical, including molecular weight, biological activity, biological source, chemical classification, and literature references. To further bridge computational insights with drug discovery, a similarity search was performed between all PPD phytochemicals and FDA-approved drugs, facilitating the identification of structurally related compounds that may advance into preclinical and clinical drug development stages (drug repurposing).

To ensure efficient data retrieval, PPD offers a comprehensive search interface, allowing users to query the database by plant name, molecular weight range, CAS ID, chemical name, and reported bioactivity. Users can perform exclusive or combined searches so as to prune the results to a selective number of phytoconstituents with respect to their associated properties. By integrating traditional ethnobotanical knowledge with computational insights, the PPD is a valuable open-access resource for identifying potential candidates for neurodegenerative diseases, bridging traditional medicine with modern science, and accelerating natural product-based drug discovery for neurological disorders.

## 3 Materials and methods

### 3.1 Data collection

The data on the psychoactive plants and their phytochemicals was obtained from four different authenticated sources viz, i) IMPPAT, ii) Dr. Duke’s Phytochemical and Ethnobotanical Databases, iii) KNApSAcK, and iv) The Dictionary of Natural Products (DNP). Duplicate entries from different sources were systematically removed. Each phytochemical was cross-validated with its corresponding reported literature. Plant names were authenticated using World Flora Online (WFO, 2023) (WFO) and further verified through MPNS [https://mpns.science.kew.org] and POWO (POWO, 2025) [https://powo.science.kew.org]. The database includes 124 psychoactive plants from 60 families, encompassing over 7,000 phytochemicals (Supplementary Table S1). Key families include Solanaceae (14 plants), Lamiaceae (9 plants), Apocynaceae (8 plants), and Fabaceae (8 plants), which are widely recognized for their neuroactive phytochemicals (Alrashedy N. A. et al., 2016; Gebhardt, 2016; Uritu C. M. et al., 2018). Solanaceae constitutes approximately 11% of the total number, with notable plants like *Nicotiana tabacum L*., whose metabolites hold therapeutic potential against Alzheimer’s disease (AD), Parkinson’s disease (PD), inflammatory diseases, obesity, and fatty liver (Zhang W. et al., 2024) (Supplementary Figures S1, S2).

Traditional medicinal knowledge of all these plants was integrated from the Traditional Knowledge Digital Library (TKDL), which revealed that 31 medicinal plants are involved in treatments for rheumatism, nervous system disorders, urinary diseases, pain, tuberculosis, and cognitive impairments (Supplementary Figure S3) (Sharma, 2017). Examples include *P*. *longum L*., *Terminalia bellirica (Gaertn*.*) Roxb*., *Cannabis sativa L*., *Datura stramonium L*., *Acorus calamus L*., and *Withania somnifera (L*.*) Dunal*, which have been historically used in Ayurvedic formulations (Choudhary N. et al., 2018; Tiwana G. et al., 2024; Odieka A.E. et al., 2022; Soni P. et al., 2012; Sharma V. et al., 2020; Mukherjee P.K. et al., 2021). Twenty-five percent of the plants in the database are reportedly used in various traditional formulations (Supplementary Figure S4), highlighting the ethnopharmacological significance of these species in guiding drug discovery (Pirintsos S. et al., 2022).

### 3.2 Data validation

To ensure accuracy, phytochemical data were cross-referenced with PubChem, DNP, KNApSAcK, Dr. Duke’s database, and IMPPAT. All structures were verified using PubChem (Kim S. et al., 2023). Unique identifiers (PPD_IDs) were assigned to each phytochemical. High-quality 2D molecular structures were generated using Schrödinger Maestro (Mumtaz A. et al., 2017). An open-source database based on MySQL, PHP, and Apache was developed with filtering capabilities for plant names, CAS IDs, molecular weights, and bioactivity data (Sharma A. et al., 2014). Analysis of the database revealed that close to 3,000 phytochemicals have molecular weight <500 g/mol (Supplementary Figure S5), thereby aligning with drug-likeness criteria. At least 1,887 phytochemicals have already been reported to have activity against multiple nervous disorders (Supplementary Figure S6). These compounds target neuroinflammation, oxidative stress, and misfolded protein aggregation. Interestingly, 4,898 phytochemicals remain unstudied for their potential in neurodegenerative diseases, highlighting opportunities for further research. More than 25% of the phytochemicals in the database exhibit activity against neurological diseases (Supplementary Figure S7). Notable examples include cannabidiol, CBD from *Cannabis sativa L*., and tropane alkaloids from *Hyoscyamus niger L*., which are known for their neuroprotective effects in preclinical and clinical studies (Stasiłowicz-Krzemień A. et al., 2024; Rehman M. U. et al., 2019; Jaremicz Z. et al., 2014).

### 3.3 Computational analysis

ADMET analysis of all the phytochemicals was conducted using QikProp (Small-Molecule, 2020), which revealed that more than 2000 phytochemicals (∼30%) fall under CNS-active criteria. These phytochemicals could be promising candidates to be taken forward as they already indicate neuroactive potential (Guan L et al.,2018). Compounds like galantamine and berberine exhibited favorable pharmacokinetics, supporting their therapeutic relevance in AD and PD (Rezaul Islam M. et al., 2024).

Molecular docking studies were conducted on NLRP3 (PDB ID: 7ALV), a neuroinflammatory target. Protein structures were refined using the Protein Preparation Wizard to optimize hydrogen bonding, loop regions, and energy minimization (Schrodinger release, 2019; Jin T. et al., 2023). Docking validation involved re-docking the co-crystallized inhibitor to ensure accurate binding-site prediction. Ligands were prepared in LigPrep (OPLS_2005 force field), and XP docking in Glide was performed for precision (Friesner R. A. et al., 2006). Among the 7,000 phytochemicals, 250 exhibited strong binding energy, with 125 scoring below –11 kcal/mol. It is pertinent to mention that the lower the binding energy, the better the target inhibition. Notably, Tellimargradin I (*Juglans regia L*.) demonstrated interactions with Ala227 and Arg578, similar to MCC950, an NLRP3 inhibitor (Supplementary Figure S8) (Blevins H. M. et al., 2022). This information could be very useful for medicinal chemists when selecting the appropriate functional groups while designing potential inhibitors based on the listed phytochemicals.

Using ClassyFire, the phytochemicals were classified into 175 chemical categories (Supplementary Table S2). Major classes include prenol lipids (25%), alkaloids (9%), flavonoids (7%), terpenoids (8%), and organooxygen compounds (7%), which are known for their neuroprotective properties (Djoumbou Feunang Y. et al., 2016; Xu B. et al., 2022).

Tanimoto similarity analysis (threshold 0.7) with the FDA-approved DrugBank library identified 371 phytochemicals with structural resemblance to approved drugs. This information could be very useful for drug repurposing (Supplementary Table S3). Morphine (100% similarity with DB00295) and caffeine exemplify natural compounds with cognitive-enhancing potential (Bajusz D. et al., 2015; Cummings J. et al., 2024).

### 3.4 Database features and applications

**FIGURE 1:**
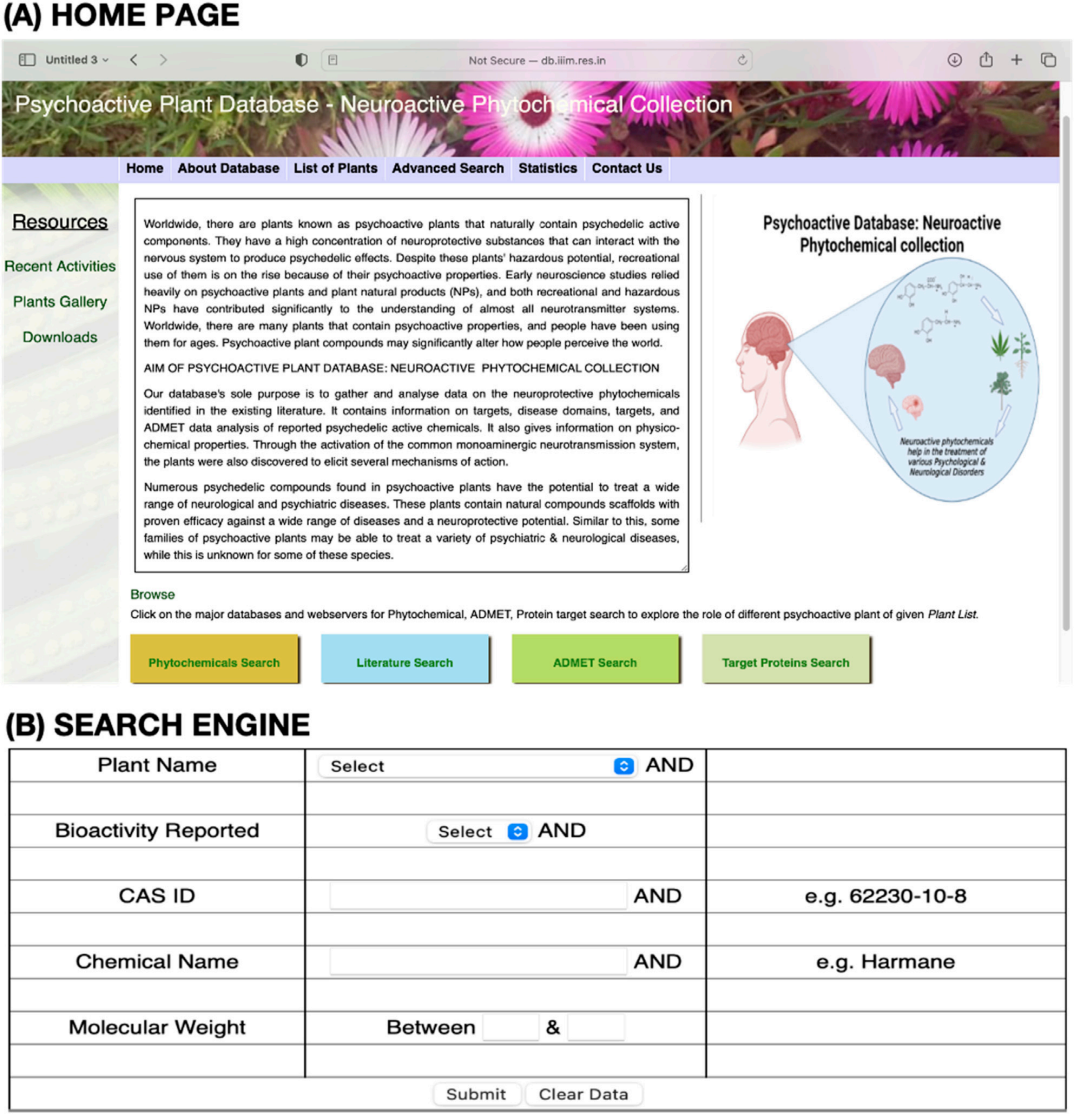
**(A)** Home page of the Psychoactive Plant Database (PPD) and **(B)** its search engine.

The PPD is a user-friendly, open-source platform designed for modern research. It features:

• Search functionalities for plant names, molecular weights, CAS IDs, bioactivity, and chemical identifiers (the international chemical identifier (InChI) and the simplified molecular input line entry system (SMILES)).
• Substructure and similarity search tools for identifying related phytochemicals.
• Plant image galleries and interactive visualizations to enhance engagement (Figure 1).
• Regular updates to integrate new research on psychoactive plants.

The PPD is a resource for identifying potential phytochemicals, integrating traditional knowledge, and facilitating drug discovery. With its structured data retrieval system, it could accelerate research in neurodegenerative diseases, ethnomedicine, and personalized medicine. Although some physicochemical properties are marked as “N/A” due to missing data, the database remains an invaluable tool for scientific discovery, bridging traditional knowledge with modern research.

### 3.5 Examples of use

**FIGURE 2:**
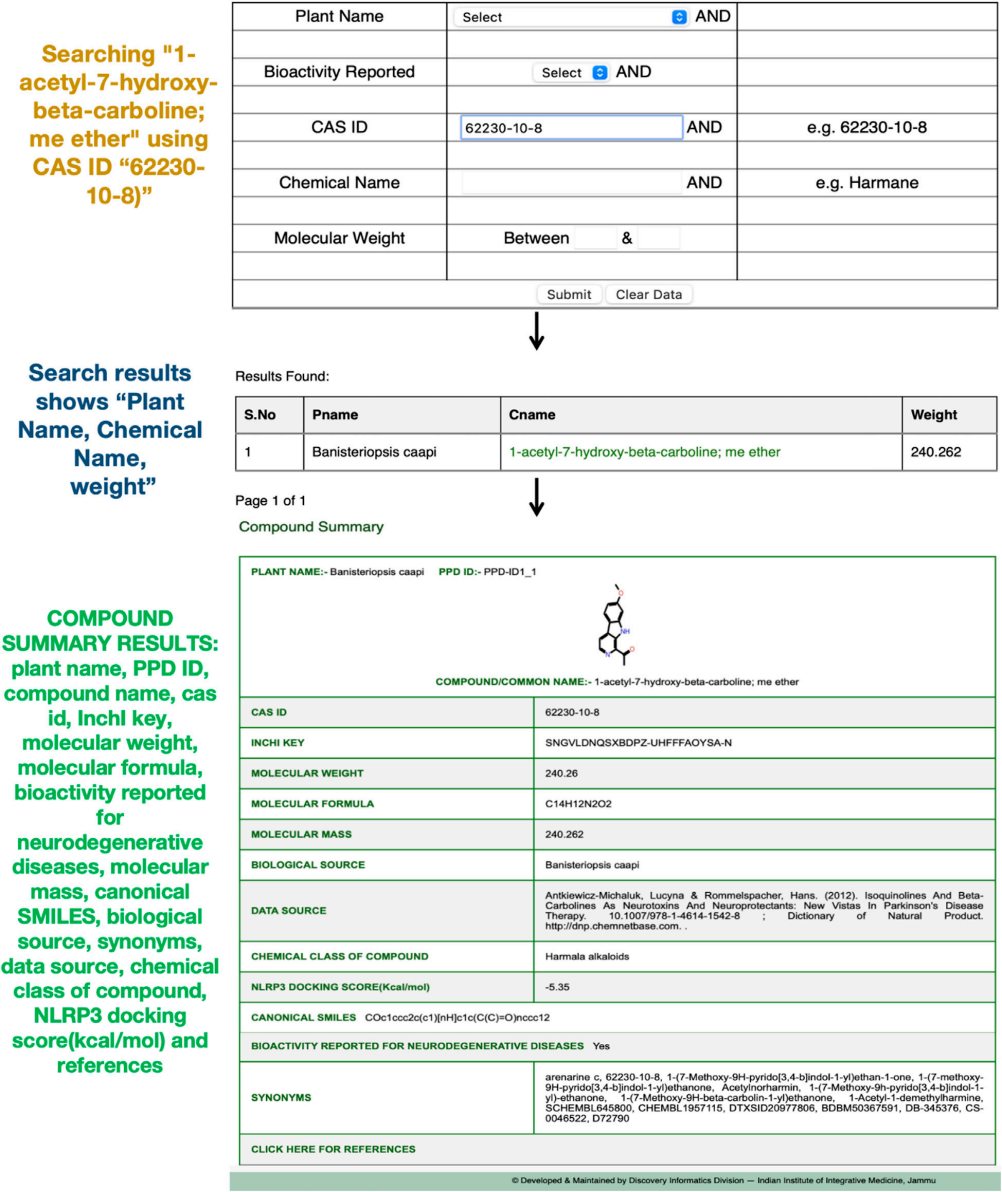
Compound search results for “1-acetyl-7-hydroxy-beta-carboline; me ether” (CAS ID: 62230-10-8) in the PPD, displaying its detailed properties, biological sources, and docking score with the NLRP3 target protein.

A search for the phytochemical “1-acetyl-7-hydroxy-beta-carboline; me ether” (CAS ID: 62230-10-8) in the PPD database retrieves comprehensive details across 16 key properties, including plant name, PPD-ID, compound/common name, CAS ID, molecular weight, InChI key, molecular formula, bioactivity reported for neurodegenerative diseases, canonical SMILES, biological source, synonyms, data source, chemical class, molecular mass, and docking score with the NLRP3 target protein, along with references (Figure 2). This phytochemical is of particular interest due to its documented neuroactive potential in various experimental studies (Beutler J. A., 2009).

Similarly, a search for “harmaline” using its chemical name retrieves its complete profile, highlighting its neuroprotective role as a reversible monoamine oxidase A (MAO-A) inhibitor, which has been studied in the context of neurological disorders (Morales-García J. A. et al., 2017). The compound summary includes all 16 properties, along with its docking score with the NLRP3 target protein, as illustrated in Supplementary Figure S9.

These search functionalities demonstrate PPD’s efficiency in enabling rapid access to essential phytochemical information, supporting research into bioactive compounds targeting neurological disorders.

## 4 Conclusion

The Psychoactive Plant Database (PPD) is a ground-breaking resource that connects modern drug discovery with traditional medicine. The database offers an extensive platform for investigating the therapeutic potential of psychoactive plants by listing more than 7,000 phytochemicals from 124 medicinal plants. It is an essential resource for researchers studying neurological and neurodegenerative disorders because it combines ethnopharmacological knowledge, computer analyses, and comprehensive phytochemical data. The medicinal potential of phytochemicals produced from plants is highlighted by important features such as molecular docking investigations, phytochemical classification, and *in silico* ADMET profiling.

*In silico* ADMET profiling, focused on CNS activity, of all the phytoconstituents was done and revealed that 29% of the compounds exhibited predicted CNS-active properties, with favorable pharmacokinetics supported by molecular weights below 500 g/mol. The database comprehends important information, such as molecular weight, biological activity, biological source, and class, for all the collected phytoconstituents. Literature references of each phytoconstituent are also compiled and documented in the database. Additionally, all the phytoconstituents were docked in the target NLRP3, which is a key target in neuroinflammation, and the binding affinity was incorporated into the database for estimating the role of these phytoconstitutents in inhibiting the NLRP3 inflammasome activity. A similarity search of all these phytoconstituents was carried out on FDA-approved drugs, the result of which could be useful for drug repurposing. The entire database is searchable by plant name and by using various keywords such as molecular weight range, CAS ID, chemical name, biological activity, and more, ensuring ease of access for researchers.

Overall, an attempt has been made to consolidate the body of traditional knowledge related to neurodegenerative diseases on a common single platform in order to accelerate neurological drug discovery based on natural products. PPD’s emphasis on traditional knowledge ensures that valuable insights from indigenous and ancient practices are preserved and used in modern research. By facilitating the discovery of novel neuroprotective agents, PPD paves the way for safer and more effective treatments for neurological disorders, addressing an urgent global need.

## 5 Future prospectives

The database provides extensive information on phytochemicals from known psychoactive plants, along with their physicochemical properties, reported bioactivity, predicted CNS properties, literature references, and predicted interactions with NLRP3, a key target for neuroinflammation. This comprehensive dataset in a single platform would be highly valuable for researchers working in the field of neurodegenerative diseases.

The PPD will be a valuable tool for medicinal chemists, facilitating structure–activity relationship (SAR) studies across diverse phytochemical classes. This resource could also be a starting point for synthesizing new compounds based on selected scaffolds for neurodegenerative disease targets. Additionally, a network pharmacology approach can be applied to identify new potential targets and compounds and explore synergistic interactions among various phytochemicals in the context of neurodegenerative diseases.

The database’s extensive search functionality allows users to retrieve phytochemicals based on physicochemical properties, biological activity, CAS ID, and other relevant parameters. The database will also be regularly updated based on new research and user inputs and may incorporate new features as per research requirements. If significant modifications are made, a new version will be released and published.

## Data Availability

The original contributions presented in the study are included in the article/Supplementary Material, further inquiries can be directed to the corresponding authors. The Psychoactive Plant Database is openly available at http://db.iiim.res.in/psychoactivedb/
