# Supplementary material for: Psychoactive Plant Database: a phytochemical resource for neurological drug discovery
Source: Front Pharmacol. 2025 May 23;16:1569127. doi: 10.3389/fphar.2025.1569127 (PMC12142401; doi:10.3389/fphar.2025.1569127)

**SUPPORTING INFORMATION**

**Psychoactive Plant Database: A Phytochemical Resource for Neurological Drug Discovery**

Harjot Kaur ^a, c^ , Monika Gupta^a, c^ , Zabeer Ahmed* ^b, c^ Amit Nargotra* ^a, c^

^a^ Discovery Informatics, NPMC Division, CSIR-Indian institute of Integrative Medicine, Canal Road, Jammu-180001, India

^b^ Pharmacology Division, CSIR-Indian institute of Integrative Medicine, Canal Road, Jammu-180001, India

^c^ Academy of Scientific and Innovative Research (AcSIR), Ghaziabad-201002, India

*** Corresponding authors**

**E-mail:** [**anargotra@iiim.res.in**](mailto:anargotra@iiim.res.in)**,** [**zahmed@iiim.res.in**](mailto:zahmed@iiim.res.in)

**Address:** Dr. Amit Nargotra, Discovery Informatics Group, NPMC Division, CSIR-Indian Institute of Integrative Medicine, Canal Road, Jammu 180001, India.

Phone no.:0191-2585028 EPAB Ext.: 269

**Address:** Dr. Zabeer Ahmed, Director, CSIR-Indian Institute of Integrative Medicine, Canal Road, Jammu 180001, India.

**Table of Contents**

**Supporting Tables**

**Table S1.** Distribution of 60 families with 124 medicinal plants in Psychoactive Plant Database (PPD) along with number of compounds identified in each plant

**Table S2.** Total phytochemicals were classified using ClassyFire into 175 major chemical classes

**Table S3.** Similarity search for database phytochemicals was performed against drugbank small molecule library

**Supporting Figures**

**Figure S1** Distribution of 60 families among 124 medicinal plants

**Figure S2** Different psychoactive plant classification, showing 11%, which is the highest classification representing the Solanaceae plant family

**Figure S3** Distribution of 31 Psychoactive plants involved in various diseases

**Figure S4** Traditional formulations for many diseases include 25% psychoactive plants

**Figure S5** Molecular weight distribution of phytochemicals in the PPD database

**Figure S6** Number of phytochemicals involved in various neurodegenerative diseases

**Figure S7** Phytochemicals involved in neurodegenerative diseases

**Figure S8** Molecular Docking analysis of phytochemicals present in the database (A) and the top molecule Tellimagradin I 3-D interaction with the NLRP3 target protein (B)

**Figure S9** Compound search for phytochemical “harmaline” with chemical name in PPD database

**Supporting Tables**

**Table S1.** Distribution of 60 families with 124 medicinal plants in Psychoactive Plant Database (PPD) along with number of compounds identified in each plant

| 1. **No.** | **Plant Name** | **Accepted Scientific names of plants** | **Plant Family Name** | **Number of Phytochemicals** |
| --- | --- | --- | --- | --- |
| 1 | *Banisteriopsis caapi* | [*Banisteriopsis caapi (Spruce ex Griseb.) C.V.Morton*](https://mpns.science.kew.org/mpns-portal/plantDetail?plantId=2668869&query=Banisteriopsis+caapi&filter=&fuzzy=false&nameType=all&dbs=wcsCmp) | Malpighiaceae | 13 |
| 2 | *Arnebia nobilis* | [*Arnebia speciosa Aitch. & Hemsl.*](https://mpns.science.kew.org/mpns-portal/plantDetail?plantId=2653025&query=Arnebia+nobilis&filter=&fuzzy=false&nameType=all&dbs=wcsCmp) | Boraginaceae | 17 |
| 3 | *Marsilea quadrifolia* | [*Marsilea quadrifolia L.*](https://mpns.science.kew.org/mpns-portal/plantDetail?plantId=3145458&query=Marsilea+quadrifolia&filter=&fuzzy=false&nameType=all&dbs=wcsCmp) | Marsileaceae | 36 |
| 4 | *Psychotria virdis* | [*Psychotria viridis Ruiz & Pav.*](https://mpns.science.kew.org/mpns-portal/plantDetail?plantId=170134&query=Psychotria+virdis&filter=&fuzzy=true&nameType=all&dbs=wcs) | Rubiaceae | 1 |
| 5 | *Juglans nigra* | [*Juglans nigra L.*](https://mpns.science.kew.org/mpns-portal/plantDetail?plantId=2331834&query=Juglans+nigra&filter=&fuzzy=false&nameType=all&dbs=wcsCmp) | Juglandaceae | 109 |
| 6 | *Illigera luzonensis* | [*Illigera luzonensis (C.Presl) Merr.*](https://mpns.science.kew.org/mpns-portal/plantDetail?plantId=2861947&query=Illigera+luzonensis&filter=&fuzzy=false&nameType=all&dbs=wcsCmp) | Hernandiaceae | 17 |
| 7 | *Scletium tortuosum (L.) N.E.Br.* | *Mesembryanthemum tortuosum L.* | Aizoaceae | 5 |
| 8 | *Mimosa tenuiflora* | [*Mimosa tenuiflora (Willd.) Poir.*](https://mpns.science.kew.org/mpns-portal/plantDetail?plantId=2374136&query=Mimosa+tenuiflora&filter=&fuzzy=false&nameType=all&dbs=wcs) | Fabaceae | 8 |
| 9 | *Lactuca virosa* | [*Lactuca virosa L.*](https://mpns.science.kew.org/mpns-portal/plantDetail?plantId=2912755&query=Lactuca+virosa&filter=&fuzzy=false&nameType=all&dbs=wcsCmp) | Asteraceae | 40 |
| 10 | *Carissa edulis* | *Carissa edulis (Forssk.) Vahl* | Apocynaceae | 16 |
| 11 | *Calea ternifolia* | [*Calea ternifolia Kunth*](https://mpns.science.kew.org/mpns-portal/plantDetail?plantId=2925874&query=Calea+ternifolia&filter=&fuzzy=false&nameType=all&dbs=wcsCmp) | Asteraceae | 5 |
| 12 | *Boswellia sacra* | [*Boswellia sacra Flück.*](https://mpns.science.kew.org/mpns-portal/plantDetail?plantId=2680579&query=Boswellia+sacra&filter=&fuzzy=false&nameType=all&dbs=wcsCmp) | Burseraceae | 213 |
| 13 | *Lobelia tupa* | [*Lobelia tupa L.*](https://mpns.science.kew.org/mpns-portal/plantDetail?plantId=353574&query=Lobelia+tupa&filter=&fuzzy=false&nameType=all&dbs=wcs) | Campanulaceae | 4 |
| 14 | *Pachycereus*  *pecten-aboriginum* | [*Pachycereus pecten-aboriginum (Engelm. ex S.Watson) Britton & Rose*](https://mpns.science.kew.org/mpns-portal/plantDetail?plantId=2414410&query=Pachycereus+pecten-aboriginum&filter=&fuzzy=false&nameType=all&dbs=wcsCmp) | Cactaceae | 1 |
| 15 | *Terminalia bellirica* | [*Terminalia bellirica (Gaertn.) Roxb.*](https://mpns.science.kew.org/mpns-portal/plantDetail?plantId=2431540&query=Terminalia+bellirica&filter=&fuzzy=false&nameType=all&dbs=wcsCmp) | Combretaceae | 83 |
| 16 | *Argyreia nervosa* | [*Argyreia nervosa (Burm.f.) Bojer*](https://mpns.science.kew.org/mpns-portal/plantDetail?plantId=483156&query=Argyreia+nervosa&filter=&fuzzy=false&nameType=all&dbs=wcs) | Convolvulaceae | 24 |
| 17 | *Turbina corymbosa* | [*Ipomoea corymbosa (L.) Roth*](https://mpns.science.kew.org/mpns-portal/plantDetail?plantId=481122&query=Turbina+corymbosa&filter=&fuzzy=false&nameType=all&dbs=wcs) | Convolvulaceae | 6 |
| 18 | *Ipomoea carnea* | [*Ipomoea carnea Jacq.*](https://mpns.science.kew.org/mpns-portal/plantDetail?plantId=480996&query=Ipomoea+carnea&filter=&fuzzy=false&nameType=all&dbs=wcs) | Convolvulaceae | 22 |
| 19 | *Juniperus recurva* | *Juniperus recurva Buch.-Ham. ex D.Don* | Cupressaceae | 104 |
| 20 | *Erythroxylum vaccinifolium* | [*Erythroxylum vacciniifolium Mart.*](https://mpns.science.kew.org/mpns-portal/plantDetail?plantId=2801719&query=Erythroxylum+vaccinifolium&filter=&fuzzy=false&nameType=all&dbs=wcsCmp) | Erythroxylaceae | 1 |
| 21 | *Anadenanthera peregrina* | [*Anadenanthera peregrina (L.) Speg.*](https://mpns.science.kew.org/mpns-portal/plantDetail?plantId=2635963&query=Anadenanthera+peregrina&filter=&fuzzy=false&nameType=all&dbs=wcs) | Fabaceae | 21 |
| 22 | *Desmanthus illinoensis* | [*Desmanthus illinoensis (Michx.) MacMill. ex B.L.Rob. & Fernald*](https://mpns.science.kew.org/mpns-portal/plantDetail?plantId=2761939&query=Desmanthus+illinoensis&filter=&fuzzy=false&nameType=all&dbs=wcs) | Fabaceae | 10 |
| 23 | *Lonchocarpus violaceus* | *Lonchocarpus violaceus (Jacq.) Kunth ex* | Fabaceae | 7 |
| 24 | *Rhynchosia pyramidalis* | *Rhynchosia pyramidalis (Lam.) Urb.* | Fabaceae | 6 |
| 25 | *Leonurus cardiaca* | *Leonurus cardiaca L.* | Lamiaceae | 134 |
| 26 | *Plectranthus scutellarioides* | *Coleus scutellarioides (L.) Benth.* | Lamiaceae | 20 |
| 27 | *Scutelleria lateriflora* | [*Scutellaria lateriflora L.*](https://mpns.science.kew.org/mpns-portal/plantDetail?plantId=189301&query=Scutelleria+lateriflora&filter=&fuzzy=true&nameType=all&dbs=wcs) | Lamiaceae | 56 |
| 28 | *Sassafras albidum* | [*Sassafras albidum (Nutt.) Nees*](https://mpns.science.kew.org/mpns-portal/plantDetail?plantId=2591964&query=Sassafras+albidum&filter=&fuzzy=false&nameType=all&dbs=wcsCmp) | Lauraceae | 124 |
| 29 | *Sida acuta* | [*Sida acuta Burm.f.*](https://mpns.science.kew.org/mpns-portal/plantDetail?plantId=2588280&query=Sida+acuta&filter=&fuzzy=false&nameType=all&dbs=wcsCmp) | Malvaceae | 34 |
| 30 | *Tilia cordata* | [*Tilia cordata Mill.*](https://mpns.science.kew.org/mpns-portal/plantDetail?plantId=2518181&query=Tilia+cordata&filter=&fuzzy=false&nameType=all&dbs=wcsCmp) | Malvaceae | 150 |
| 31 | *Osteophloeum platyspermum* | [*Osteophloeum platyspermum (Spruce ex A.DC.) Warb.*](https://mpns.science.kew.org/mpns-portal/plantDetail?plantId=2397780&query=Osteophloeum+platyspermum&filter=&fuzzy=false&nameType=all&dbs=wcsCmp) | Myristicaceae | 6 |
| 32 | *Nymphaea candida* | *Nymphaea candida C.Presl* | Nymphaeaceae | 2 |
| 33 | *Vanilla planifolia* | *Vanilla planifolia Andrews* | Orchidaceae | 229 |
| 34 | *Cistanche deserticola* | [*Cistanche deserticola Ma*](https://mpns.science.kew.org/mpns-portal/plantDetail?plantId=2723155&query=Cistanche+deserticola&filter=&fuzzy=false&nameType=all&dbs=wcsCmp) | Orobanchaceae | 23 |
| 35 | *Meconopsis horridula* | *Meconopsis horridula Hook.f. & Thomson* | Papaveraceae | 11 |
| 36 | *Turnera diffusa* | *Turnera diffusa Willd. ex Schult.* | Turneraceae | 93 |
| 37 | *Lolium temulentum* | *Lolium temulentum L.* | Poaceae | 25 |
| 38 | *Catunaregam nilotica* | *Catunaregam nilotica (Stapf) Tirveng.* | Rubiaceae | 2 |
| 39 | *Corynanthe pachyceras* | *Corynanthe pachyceras K.Schum.* | Rubiaceae | 1 |
| 40 | *Paullinia cupana* | *Paullinia cupana Kunth* | Sapindaceae | 28 |
| 41 | *Brugmansia suaveolens* | [*Brugmansia suaveolens (Humb. & Bonpl. ex Willd.) Sweet*](https://mpns.science.kew.org/mpns-portal/plantDetail?plantId=2684019&query=Brugmansia+suaveolens&filter=&fuzzy=false&nameType=all&dbs=wcsCmp) | Solanaceae | 13 |
| 42 | *Brunfelsia grandiflora* | [*Brunfelsia grandiflora D.Don*](https://mpns.science.kew.org/mpns-portal/plantDetail?plantId=2684099&query=Brunfelsia+grandiflora&filter=&fuzzy=false&nameType=all&dbs=wcsCmp) | Solanaceae | 9 |
| 43 | *Cestrum diurnum* | [*Cestrum diurnum L.*](https://mpns.science.kew.org/mpns-portal/plantDetail?plantId=2713430&query=Cestrum+diurnum&filter=&fuzzy=false&nameType=all&dbs=wcsCmp) | Solanaceae | 12 |
| 44 | *Lochroma fuchsioides* | [*Iochroma fuchsioides (Bonpl.) Miers*](https://mpns.science.kew.org/mpns-portal/plantDetail?plantId=2866020&query=Lochroma+fuchsioides&filter=&fuzzy=true&nameType=all&dbs=wcsCmp) | Solanaceae | 3 |
| 45 | *Mandragora officinarum* | [*Mandragora officinarum L.*](https://mpns.science.kew.org/mpns-portal/plantDetail?plantId=2506563&query=Mandragora+officinarum&filter=&fuzzy=false&nameType=all&dbs=wcsCmp) | Solanaceae | 28 |
| 46 | *Cannabis sativa* | [*Cannabis sativa L.*](https://mpns.science.kew.org/mpns-portal/plantDetail?plantId=2696480&query=Cannabis+sativa&filter=&fuzzy=false&nameType=all&dbs=wcsCmp) | Cannabaceae | 479 |
| 47 | *Schinus terebinthifolius* | [*Schinus terebinthifolia Raddi*](https://mpns.science.kew.org/mpns-portal/plantDetail?plantId=2480191&query=Schinus+terebinthifolius&filter=&fuzzy=false&nameType=all&dbs=wcsCmp) | Anacardiaceae | 102 |
| 48 | *Juglans regia* | [*Juglans regia L.*](https://mpns.science.kew.org/mpns-portal/plantDetail?plantId=2331747&query=Juglans+regia&filter=&fuzzy=false&nameType=all&dbs=wcsCmp) | Juglandaceae | 343 |
| 49 | *Salvia divinorum* | [*Salvia divinorum Epling & Játiva*](https://mpns.science.kew.org/mpns-portal/plantDetail?plantId=182532&query=Salvia+divinorum&filter=&fuzzy=false&nameType=all&dbs=wcs) | Lamiaceae | 23 |
| 50 | *Tabernanth iboga* | [*Tabernanthe iboga Baill.*](https://mpns.science.kew.org/mpns-portal/plantDetail?plantId=200996&query=Tabernanth+iboga&filter=&fuzzy=true&nameType=all&dbs=wcs) | Apocynaceae | 30 |
| 51 | *Picralima nitida* | [*Picralima nitida (Stapf) T.Durand & H.Durand*](https://mpns.science.kew.org/mpns-portal/plantDetail?plantId=156030&query=Picralima+nitida&filter=&fuzzy=false&nameType=all&dbs=wcs) | Apocynaceae | 17 |
| 52 | *Mitragyna speciosa* | [*Mitragyna speciosa Korth.*](https://mpns.science.kew.org/mpns-portal/plantDetail?plantId=128805&query=Mitragyna+speciosa&filter=&fuzzy=false&nameType=all&dbs=wcs) | Rubiaceae | 32 |
| 53 | *Bacopa monierri* | [*Bacopa monnieri (L.) Wettst.*](https://mpns.science.kew.org/mpns-portal/plantDetail?plantId=2667648&query=Bacopa+monnieri&filter=&fuzzy=false&nameType=all&dbs=wcsCmp) | Scrophulariaceae | 32 |
| 54 | *Ginkgo biloba* | [*Ginkgo biloba L.*](https://mpns.science.kew.org/mpns-portal/plantDetail?plantId=334053&query=Ginkgo+biloba&filter=&fuzzy=false&nameType=all&dbs=wcs) | Ginkgoaceae | 105 |
| 55 | *Datura stramonium* | [*Datura stramonium L.*](https://mpns.science.kew.org/mpns-portal/plantDetail?plantId=2757848&query=Datura+stramonium&filter=&fuzzy=false&nameType=all&dbs=wcsCmp) | Solanaceae | 26 |
| 56 | *Piper methysticum* | *Piper methysticum G.Forst.* | Piperaceae | 39 |
| 57 | *Catha edulis* | *Catha edulis (Vahl) Endl.* | Celastraceae | 32 |
| 58 | *Valeriana officinalis* | *Valeriana officinalis L.* | Caprifoliaceae | 101 |
| 59 | *Mimosa pudica* | *Mimosa pudica L.* | Fabaceae | 19 |
| 60 | *Achillea milefolium* | [*Achillea millefolium L.*](https://mpns.science.kew.org/mpns-portal/plantDetail?plantId=2908230&query=Achillea+milefolium&filter=&fuzzy=false&nameType=all&dbs=wcsCmp) | Asteraceae | 146 |
| 61 | *Salvia officinalis* | [*Salvia officinalis L.*](https://mpns.science.kew.org/mpns-portal/plantDetail?plantId=183353&query=Salvia+officinalis&filter=&fuzzy=false&nameType=all&dbs=wcs) | Lamiaceae | 52 |
| 62 | *Justicia pectoralis* | *Justicia pectoralis Jacq.* | Acanthaceae | 5 |
| 63 | *Acorus calamus* | *Acorus calamus L.* | Acoraceae | 78 |
| 64 | *Angelica sinensis* | [*Angelica sinensis (Oliv.) Diels*](https://mpns.science.kew.org/mpns-portal/plantDetail?plantId=2639272&query=Angelica+sinensis&filter=&fuzzy=false&nameType=all&dbs=wcsCmp) | Apiaceae | 47 |
| 65 | *Centella asiatica* | [*Centella asiatica (L.) Urb.*](https://mpns.science.kew.org/mpns-portal/plantDetail?plantId=2708815&query=Centella+asiatica&filter=&fuzzy=false&nameType=all&dbs=wcsCmp) | Apiaceae | 53 |
| 66 | *Alstonia scholaris* | [*Alstonia scholaris (L.) R.Br.*](https://mpns.science.kew.org/mpns-portal/plantDetail?plantId=7103&query=Alstonia+scholaris&filter=&fuzzy=false&nameType=all&dbs=wcs) | Apocynaceae | 130 |
| 67 | *Apocynum venetum* | *Apocynum venetum L.* | Apocynaceae | 23 |
| 68 | *Rauvolfia serpentina* | [*Rauvolfia serpentina (L.) Benth. ex Kurz*](https://mpns.science.kew.org/mpns-portal/plantDetail?plantId=176968&query=Rauvolfia+serpentina+&filter=&fuzzy=false&nameType=all&dbs=wcs) | Apocynaceae | 17 |
| 69 | *Tabernaemontana divaricata* | [*Tabernaemontana divaricata (L.) R.Br. ex Roem. & Schult.*](https://mpns.science.kew.org/mpns-portal/plantDetail?plantId=200675&query=Tabernaemontana+divaricata&filter=&fuzzy=false&nameType=all&dbs=wcs) | Apocynaceae | 44 |
| 70 | *Voacanga africana* | [*Voacanga africana Stapf*](https://mpns.science.kew.org/mpns-portal/plantDetail?plantId=213939&query=Voacanga+africana+&filter=&fuzzy=false&nameType=all&dbs=wcs) | Apocynaceae | 46 |
| 71 | *Ilex paraguariensis* | *Ilex paraguariensis A.St.-Hil.* | Aquifoliaceae | 14 |
| 72 | *Panax ginseng* | *Panax ginseng C.A.Mey.* | Araliaceae | 306 |
| 73 | *Areca catechu* | [*Areca catechu L.*](https://mpns.science.kew.org/mpns-portal/plantDetail?plantId=14517&query=Areca+catechu&filter=&fuzzy=false&nameType=all&dbs=wcs) | Arecaceae | 18 |
| 74 | *Artemisia annua* | [*Artemisia annua L.*](https://mpns.science.kew.org/mpns-portal/plantDetail?plantId=2901442&query=Artemisia+annua&filter=&fuzzy=false&nameType=all&dbs=wcsCmp) | Compositae | 107 |
| 75 | *Tagetes erecta* | [*Tagetes erecta L.*](https://mpns.science.kew.org/mpns-portal/plantDetail?plantId=2929143&query=Tagetes+erecta&filter=&fuzzy=false&nameType=all&dbs=wcsCmp) | Compositae | 12 |
| 76 | *Lophophora williamsii* | *Lophophora williamsii (Lem. ex J.F.Cels) J.M.Coult.* | Cactaceae | 52 |
| 77 | *Humulus lupulus* | *Humulus lupulus L.* | Cannabaceae | 201 |
| 78 | *Nardostachys jatamansi* | [*Nardostachys jatamansi (D.Don) DC.*](https://mpns.science.kew.org/mpns-portal/plantDetail?plantId=2382349&query=Nardostachys+jatamansi&filter=&fuzzy=false&nameType=all&dbs=wcsCmp) | Caprifoliaceae | 44 |
| 79 | *Desfontainia spinosa* | [*Desfontainia spinosa Ruiz & Pav.*](https://mpns.science.kew.org/mpns-portal/plantDetail?plantId=514148&query=Desfontainia+spinosa+&filter=&fuzzy=false&nameType=all&dbs=wcs) | Columelliaceae | 13 |
| 80 | *Ephedra sinica* | [*Ephedra sinica Stapf*](https://mpns.science.kew.org/mpns-portal/plantDetail?plantId=333041&query=Ephedra+sinica&filter=&fuzzy=false&nameType=all&dbs=wcs) | Ephedraceae | 34 |
| 81 | *Ledum palustre* | *Ledum palustre L.* | Ericaceae | 25 |
| 82 | *Rhododendron molle* | *Rhododendron molle (Blume) G.Don* | Ericaceae | 92 |
| 83 | *Acacia nilotica* | *Acacia nilotica (L.) Willd. ex Delile* | Leguminosae | 15 |
| 84 | *Astragalus mongholicus* | *Astragalus mongholicus Bunge* | Fabaceae | 10 |
| 85 | *Calliandra anomala* | *Calliandra anomala (Kunth) J.F.Macbr.* | Fabaceae | 15 |
| 86 | *Erythrina variegata* | *Erythrina variegata L.* | Leguminosae | 84 |
| 87 | *Mucuna pruriens* | *Mucuna pruriens (L.) DC.* | Leguminosae | 27 |
| 88 | *Sophora secundiflora* | *Sophora secundiflora (Ortega) Lag. ex DC.* | Leguminosae | 15 |
| 89 | *Hypericum perforatum* | *Hypericum perforatum L.* | Hypericaceae | 101 |
| 90 | *Crocus sativus* | *Crocus sativus L.* | Iridaceae | 50 |
| 91 | *Lavandula augustifolia* | [*Lavandula angustifolia Mill.*](https://mpns.science.kew.org/mpns-portal/plantDetail?plantId=108971&query=Lavandula+augustifolia+&filter=&fuzzy=true&nameType=all&dbs=wcs) | Lamiaceae | 30 |
| 92 | *Leonotis leonurus* | [*Leonotis leonurus (L.) R.Br.*](https://mpns.science.kew.org/mpns-portal/plantDetail?plantId=109449&query=Leonotis+leonurus+&filter=&fuzzy=false&nameType=all&dbs=wcs) | Lamiaceae | 19 |
| 93 | *Melissa officinalis* | [*Melissa officinalis L.*](https://mpns.science.kew.org/mpns-portal/plantDetail?plantId=124103&query=Melissa+officinalis&filter=&fuzzy=false&nameType=all&dbs=wcs) | Lamiaceae | 31 |
| 94 | *Rosmarinus officinalis* | *Rosmarinus officinalis L.* | Lamiaceae | 48 |
| 95 | *Cinnamomum camphora* | *Cinnamomum camphora (L.) J.Presl* | Lauraceae | 27 |
| 96 | [*Strychnos nux-vomica*](https://www.google.com/search?client=safari&sca_esv=01fbc1f80832a702&rls=en&q=Strychnos+nux-vomica&spell=1&sa=X&ved=2ahUKEwih7MO5vtGLAxV3ma8BHZJOH4UQkeECKAB6BAgWEAE) | [*Strychnos nux-vomica L.*](https://mpns.science.kew.org/mpns-portal/plantDetail?plantId=2598138&query=Strychnos+nux-vomica&filter=&fuzzy=false&nameType=all&dbs=wcsCmp) | Loganiaceae | 66 |
| 97 | *Heimia salicifolia* | [*Heimia salicifolia Link*](https://mpns.science.kew.org/mpns-portal/plantDetail?plantId=2841927&query=Heimia+salicifolia+&filter=&fuzzy=false&nameType=all&dbs=wcsCmp) | Lythraceae | 34 |
| 98 | *Theobroma cacao* | [*Theobroma cacao L.*](https://mpns.science.kew.org/mpns-portal/plantDetail?plantId=2519807&query=Theobroma+cacao&filter=&fuzzy=false&nameType=all&dbs=wcsCmp) | Malvaceae | 20 |
| 99 | *Veratrum album* | [*Veratrum album L.*](https://mpns.science.kew.org/mpns-portal/plantDetail?plantId=291244&query=Veratrum+album&filter=&fuzzy=false&nameType=all&dbs=wcs) | Melanthiaceae | 55 |
| 100 | *Myristica fragrans* | [*Myristica fragrans Houtt.*](https://mpns.science.kew.org/mpns-portal/plantDetail?plantId=2500629&query=Myristica+fragrans&filter=&fuzzy=false&nameType=all&dbs=wcsCmp) | Myristicaceae | 103 |
| 101 | *Virola elongata* | [*Virola elongata (Benth.) Warb.*](https://mpns.science.kew.org/mpns-portal/plantDetail?plantId=2452056&query=Virola+elongata&filter=&fuzzy=false&nameType=all&dbs=wcsCmp) | Myristicaceae | 34 |
| 102 | *Psidium guajava* | [*Psidium guajava L.*](https://mpns.science.kew.org/mpns-portal/plantDetail?plantId=166741&query=Psidium+guajava&filter=&fuzzy=false&nameType=all&dbs=wcs) | Myrtaceae | 111 |
| 103 | *Peganum harmala* | [*Peganum harmala L.*](https://mpns.science.kew.org/mpns-portal/plantDetail?plantId=2548698&query=Peganum+harmala+&filter=&fuzzy=false&nameType=all&dbs=wcsCmp) | Nitrariaceae | 81 |
| 104 | *Ptychopetalum olacoides* | [*Ptychopetalum olacoides Benth.*](https://mpns.science.kew.org/mpns-portal/plantDetail?plantId=2552763&query=Ptychopetalum+olacoides&filter=&fuzzy=false&nameType=all&dbs=wcsCmp) | Olacaceae | 20 |
| 105 | *Pandanus amaryllifolius* | [*Pandanus amaryllifolius Roxb. ex Lindl.*](https://mpns.science.kew.org/mpns-portal/plantDetail?plantId=285639&query=Pandanus+amaryllifolius&filter=&fuzzy=false&nameType=all&dbs=wcs) | Pandanaceae | 43 |
| 106 | *Argemone mexicana* | [*Argemone mexicana L.*](https://mpns.science.kew.org/mpns-portal/plantDetail?plantId=2650509&query=Argemone+mexicana&filter=&fuzzy=false&nameType=all&dbs=wcsCmp) | Papaveraceae | 27 |
| 107 | *Eschscholzia californica* | [*Eschscholzia californica Cham.*](https://mpns.science.kew.org/mpns-portal/plantDetail?plantId=2801972&query=Eschscholzia+californica&filter=&fuzzy=false&nameType=all&dbs=wcsCmp) | Papaveraceae | 27 |
| 108 | *Papaver somniferum* | [*Papaver somniferum L.*](https://mpns.science.kew.org/mpns-portal/plantDetail?plantId=2561497&query=Papaver+somniferum&filter=&fuzzy=false&nameType=all&dbs=wcsCmp) | Papaveraceae | 64 |
| 109 | *Passiflora edulis* | [*Passiflora edulis Sims*](https://mpns.science.kew.org/mpns-portal/plantDetail?plantId=2559735&query=Passiflora+edulis&filter=&fuzzy=false&nameType=all&dbs=wcsCmp) | Passifloraceae | 79 |
| 110 | *Phytolacca acinosa* | [*Phytolacca acinosa Roxb.*](https://mpns.science.kew.org/mpns-portal/plantDetail?plantId=2562797&query=Phytolacca+acinosa&filter=&fuzzy=false&nameType=all&dbs=wcsCmp) | Phytolaccaceae | 31 |
| 111 | *Arundo donax* | [*Arundo donax L.*](https://mpns.science.kew.org/mpns-portal/plantDetail?plantId=396629&query=Arundo+donax+&filter=&fuzzy=false&nameType=all&dbs=wcs) | Poaceae | 20 |
| 112 | *Piper longum* | [*Piper longum L.*](https://mpns.science.kew.org/mpns-portal/plantDetail?plantId=2568895&query=Piper+longum&filter=&fuzzy=false&nameType=all&dbs=wcsCmp) | Piperaceae | 48 |
| 113 | *Aconitum napellus* | [*Aconitum napellus L.*](https://mpns.science.kew.org/mpns-portal/plantDetail?plantId=2619110&query=Aconitum+napellus&filter=&fuzzy=false&nameType=all&dbs=wcsCmp) | Ranunculaceae | 32 |
| 114 | *Hydrastis canadensis* | [*Hydrastis canadensis L.*](https://mpns.science.kew.org/mpns-portal/plantDetail?plantId=2855852&query=Hydrastis+canadensis&filter=&fuzzy=false&nameType=all&dbs=wcsCmp) | Ranunculaceae | 19 |
| 115 | *Coffea arabica* | [*Coffea arabica L.*](https://mpns.science.kew.org/mpns-portal/plantDetail?plantId=45400&query=Coffea+arabica&filter=&fuzzy=false&nameType=all&dbs=wcs) | Rubiaceae | 45 |
| 116 | *Atropa belladonna* | [*Atropa bella-donna L.*](https://mpns.science.kew.org/mpns-portal/plantDetail?plantId=2665943&query=Atropa+belladonna+L.&filter=&fuzzy=false&nameType=all&dbs=wcsCmp) | Solanaceae | 17 |
| 117 | *Duboisia myoporoides* | [*Duboisia myoporoides R.Br.*](https://mpns.science.kew.org/mpns-portal/plantDetail?plantId=2778803&query=Duboisia+myoporoides+&filter=&fuzzy=false&nameType=all&dbs=wcsCmp) | Solanaceae | 15 |
| 118 | *Hyoscyamus niger* | [*Hyoscyamus niger L.*](https://mpns.science.kew.org/mpns-portal/plantDetail?plantId=2857441&query=Hyoscyamus+niger&filter=&fuzzy=false&nameType=all&dbs=wcsCmp) | Solanaceae | 37 |
| 119 | *Nicotiana tobacum* | [*Nicotiana tabacum L.*](https://mpns.science.kew.org/mpns-portal/plantDetail?plantId=2382275&query=Nicotiana+tobacum&filter=&fuzzy=false&nameType=all&dbs=wcsCmp) | Solanaceae | 340 |
| 120 | *Physalis peruviana* | [*Physalis peruviana L.*](https://mpns.science.kew.org/mpns-portal/plantDetail?plantId=2549655&query=Physalis+peruviana&filter=&fuzzy=false&nameType=all&dbs=wcsCmp) | Solanaceae | 50 |
| 121 | *Solanum torvum* | *Solanum torvum Buch.-Ham. ex Wall.* | Solanaceae | 53 |
| 122 | *Withania somnifera* | [*Withania somnifera (L.) Dunal*](https://mpns.science.kew.org/mpns-portal/plantDetail?plantId=2465599&query=Withania+somnifera&filter=&fuzzy=false&nameType=all&dbs=wcsCmp) | Solanaceae | 162 |
| 123 | *[Scopolia carniolica](https://en.wikipedia.org/wiki/Scopolia_carniolica)* | [*Scopolia carniolica Jacq.*](https://mpns.science.kew.org/mpns-portal/plantDetail?plantId=2579708&query=Scopolia+carniolica&filter=&fuzzy=false&nameType=all&dbs=wcsCmp) | Solanaceae | 11 |
| 124 | *Camella sinensis* | [*Camellia sinensis (L.) Kuntze*](https://mpns.science.kew.org/mpns-portal/plantDetail?plantId=2694880&query=Camella+sinensis&filter=&fuzzy=true&nameType=all&dbs=wcsCmp) | Theaceae | 307 |

**Table S2.** Total phytochemicals were classified using ClassyFire into 175 major chemical classes

| **Class** | **No. of phytochemicals** |
| --- | --- |
| Harmala alkaloids | 11 |
| Pyrrolidines | 13 |
| Indoles and derivatives | 126 |
| Fatty Acyls | 255 |
| Steroids and steroid derivatives | 361 |
| Prenol lipids | 1755 |
| Flavonoids | 616 |
| Phenols | 98 |
| 2-arylbenzofuran flavonoids | 53 |
| Heteroaromatic compounds | 13 |
| Organooxygen compounds | 489 |
| Benzene and substituted derivatives | 257 |
| Lactones | 63 |
| Naphthalenes | 72 |
| Pyrans | 120 |
| Carboxylic acids and derivatives | 106 |
| Diazinanes | 2 |
| Dihydrofurans | 33 |
| Indoles and derivatives | 120 |
| Naphthalenes | 72 |
| Saturated hydrocarbons | 380 |
| Cinnamic acids and derivatives | 67 |
| Tannins | 47 |
| Diazines | 5 |
| Aporphines | 29 |
| Quinolines and derivatives | 21 |
| Diarylheptanoids | 2 |
| Coumarins and derivatives | 40 |
| Lactones | 63 |
| Morphinans | 10 |
| Tropane alkaloids | 31 |
| Unsaturated hydrocarbons | 37 |
| Hydroxy acids and derivatives | 11 |
| Dibenzylbutane lignans | 8 |
| Furanoid lignans | 50 |
| Organic oxides | 8 |
| Phenol esters | 3 |
| Epoxides | 18 |
| Tetrahydrofurans | 15 |
| Oxanes | 21 |
| Sulfoxides | 1 |
| Phenol ethers | 29 |
| Tetrahydroisoquinolines | 29 |
| Glycerophospholipids | 3 |
| Lignans, neolignans and related compounds | 29 |
| Ergoline and derivatives | 20 |
| Strychnos alkaloids | 65 |
| Tropones | 2 |
| Stilbenes | 27 |
| Isoflavonoids | 89 |
| Pyrans | 15 |
| Benzopyrans | 94 |
| Dihydrofurans | 33 |
| Benzodioxoles | 41 |
| Isoquinolines and derivatives | 23 |
| Organonitrogen compounds | 15 |
| Diazanaphthalenes | 22 |
| Benzofurans | 27 |
| Tetrapyrroles and derivatives | 4 |
| Aryltetralin lignans | 13 |
| Furans | 3 |
| Benzothiazoles | 1 |
| Cinnamaldehydes | 4 |
| Cinnamyl alcohols | 4 |
| Oxolanes | 2 |
| Lignan glycosides | 5 |
| Rhoeadine alkaloids | 5 |
| Imidazopyrimidines | 25 |
| Polycyclic hydrocarbons | 1 |
| Loline alkaloids and derivatives | 4 |
| Purine nucleosides | 2 |
| Alkaloids and derivatives | 32 |
| Pyrroles | 2 |
| Pyridines and derivatives | 40 |
| Oxazinanes | 2 |
| Oxazepines | 1 |
| Macrolactams | 2 |
| 2-arylbenzofuran flavonoids | 1 |
| Indanes | 15 |
| Arylnaphthalene lignans | 2 |
| Piperidines | 20 |
| Phenanthrenes and derivatives | 9 |
| Homogeneous halogens | 1 |
| Transition metal organides | 1 |
| Linear 1,3-diarylpropanoids | 42 |
| Saccharolipids | 7 |
| Protoberberine alkaloids and derivatives | 7 |
| Tetralins | 3 |
| Pteridines and derivatives | 2 |
| Biotin and derivatives | 2 |
| Ibogan-type alkaloids | 17 |
| Quinolizidines | 25 |
| Quebrachamine alkaloids | 5 |
| Yohimbine alkaloids | 13 |
| Corynanthean-type alkaloids | 19 |
| Quinolizines | 1 |
| Indolizidines | 6 |
| Isobenzofurans | 12 |
| Indolonaphthyridine alkaloids | 2 |
| Kavalactones | 17 |
| Cyclobutane lignans | 5 |
| Macrolides and analogues | 8 |
| Dioxanes | 3 |
| Homogeneous alkaline earth metal compounds | 2 |
| Homogeneous other non-metal compounds | 2 |
| Depsides and depsidones | 1 |
| 3,4-dihydrocoumarins | 1 |
| Keto acids and derivatives | 3 |
| Isocoumarans | 21 |
| Vallesaman alkaloids | 4 |
| Rhazinilam alkaloids | 1 |
| Pleiocarpaman alkaloids | 2 |
| Akuammilan and related alkaloids | 1 |
| Ajmaline-sarpagine alkaloids | 1 |
| Aspidospermatan-type alkaloids | 16 |
| Vobasan alkaloids | 2 |
| Eburnan-type alkaloids | 4 |
| Plumeran-type alkaloids | 4 |
| Halohydrins | 2 |
| Pyrroloazepines | 1 |
| Glycerolipids | 2 |
| Coumarans | 4 |
| Oxepanes | 7 |
| Dioxolopyrans | 2 |
| Organic hydroperoxides | 2 |
| Bi- and oligothiophenes | 1 |
| Dihydroisoquinolines | 2 |
| Furopyrroles | 1 |
| Organic trisulfides | 1 |
| Thiiranes | 2 |
| Triphenyl compounds | 1 |
| Carbene-type 1,3-dipolar compounds | 4 |
| Thioethers | 3 |
| Homoisoflavonoids | 2 |
| Naphthopyrans | 5 |
| Naphthofurans | 8 |
| Erythrina alkaloids | 22 |
| Lupin alkaloids | 4 |
| Benzodioxanes | 2 |
| Pyrenes | 3 |
| Perylenequinones | 2 |
| Anthracenes | 14 |
| Furopyrans | 2 |
| Phenylpropanoic acids | 3 |
| Benzoxepines | 5 |
| Fluorenes | 3 |
| Phenanthrolines | 1 |
| Neoflavonoids | 26 |
| Thiols | 4 |
| Pyrrolines | 3 |
| Stemona alkaloids | 1 |
| Azaspirodecane derivatives | 5 |
| Benzophenanthridine alkaloids | 5 |
| Protopine alkaloids | 11 |
| Isoindoles and derivatives | 6 |
| Phthalide isoquinolines | 7 |
| Dibenzazecins | 1 |
| Dioxepanes | 1 |
| Thiocarboxylic acids and derivatives | 1 |
| Oxathianes | 1 |
| Benzoxazines | 2 |
| Aristolactams | 3 |
| Carboximidic acids and derivatives | 1 |
| Alkaloids and derivatives | 18 |
| Benzenoids | 1 |
| Coumarinolignans | 5 |
| Organometalloid compounds | 4 |
| Isocoumarins and derivatives | 5 |
| Thioselenides | 1 |
| Indenes and isoindenes | 2 |
| Benzopyrans | 96 |
| Benzopyrazoles | 1 |
| Azoles | 2 |
| Azolidines | 1 |
| Organic sulfuric acids and derivatives | 1 |

**Supporting Figures.**

**Figure S1.** Distribution of 60 families among 124 medicinal plant

**Figure S2.** Different psychoactive plant classification, showing 11%, which is the highest classification representing the Solanaceae plant family


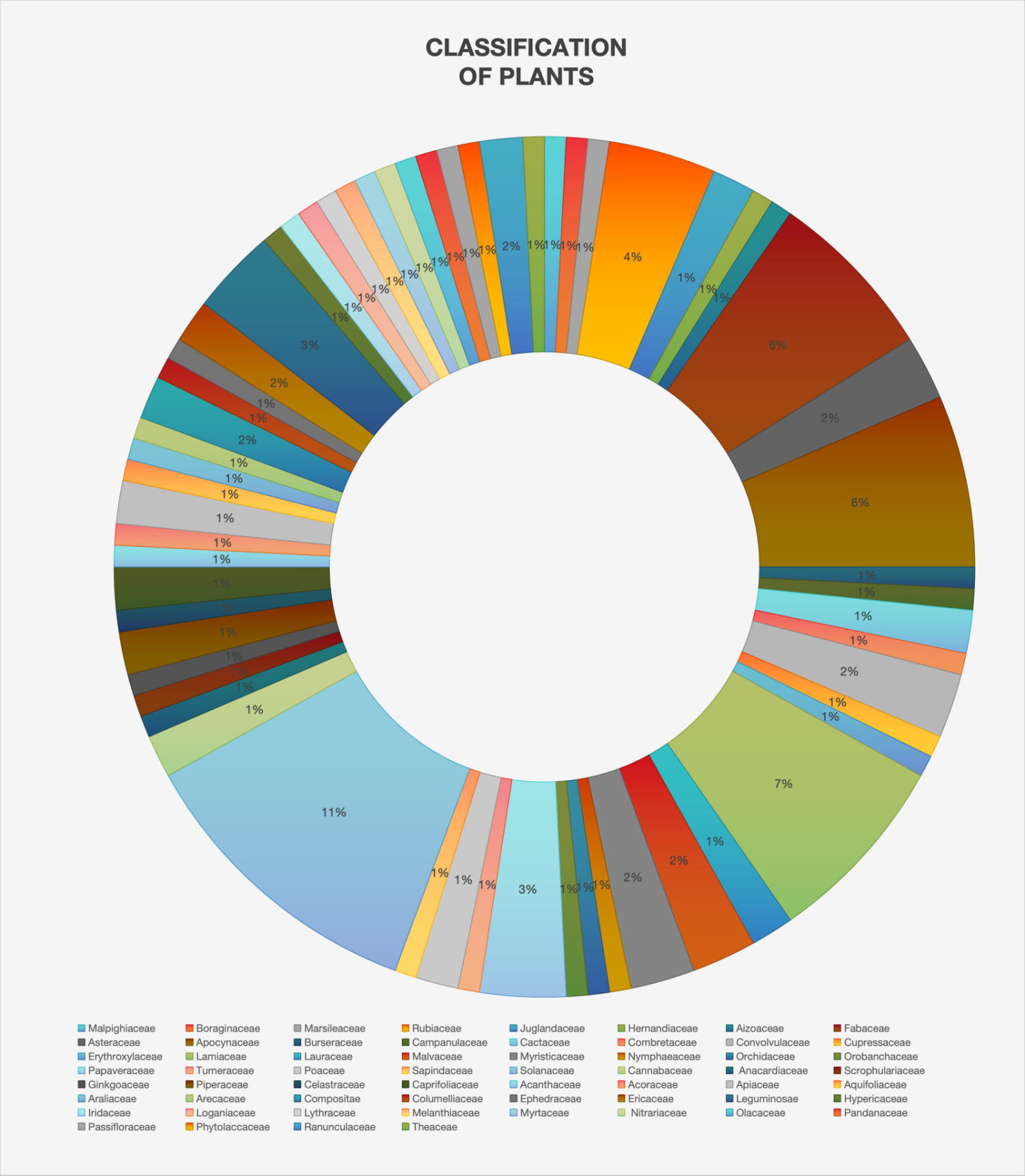


**Figure S3.** Distribution of 31 Psychoactive plants involved in various diseases

**Figure S4.** Traditional formulations for many diseases include 25% psychoactive plants

**Figure S5.** Molecular weight distribution of phytochemicals in the PPD database

**Figure S6**. Number of phytochemicals involved in various neurodegenerative disease

**Figure S7.** Phytochemicals involved in neurodegenerative diseases

**Figure S8.** Molecular Docking analysis of phytochemicals present in the database (A) and the top molecule Tellimagradin I 3-D interaction with the NLRP3 target protein (B)


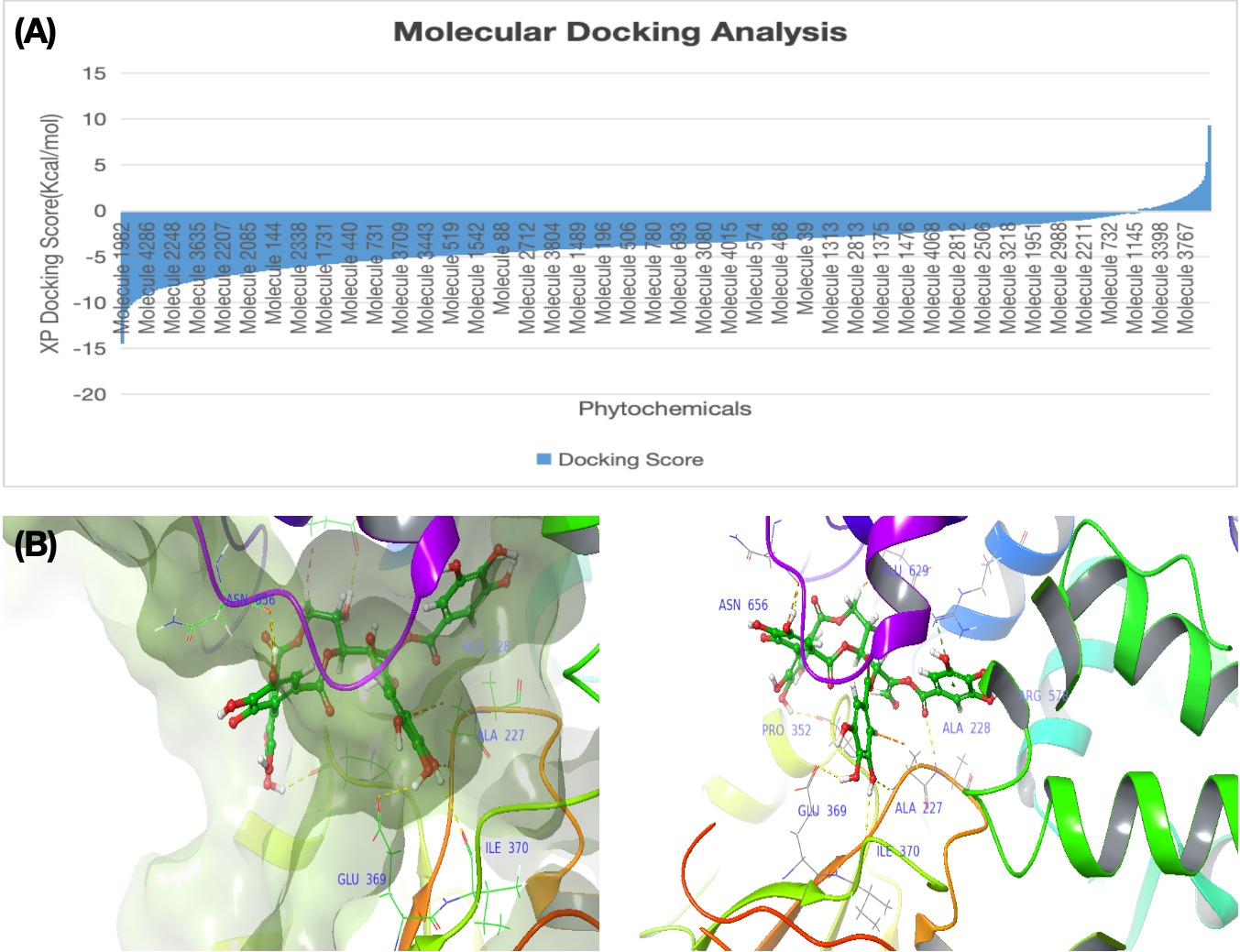


**Figure S9.** Compound search for phytochemical “harmaline” with chemical name in PPD database (fields curtailed here for clarity)


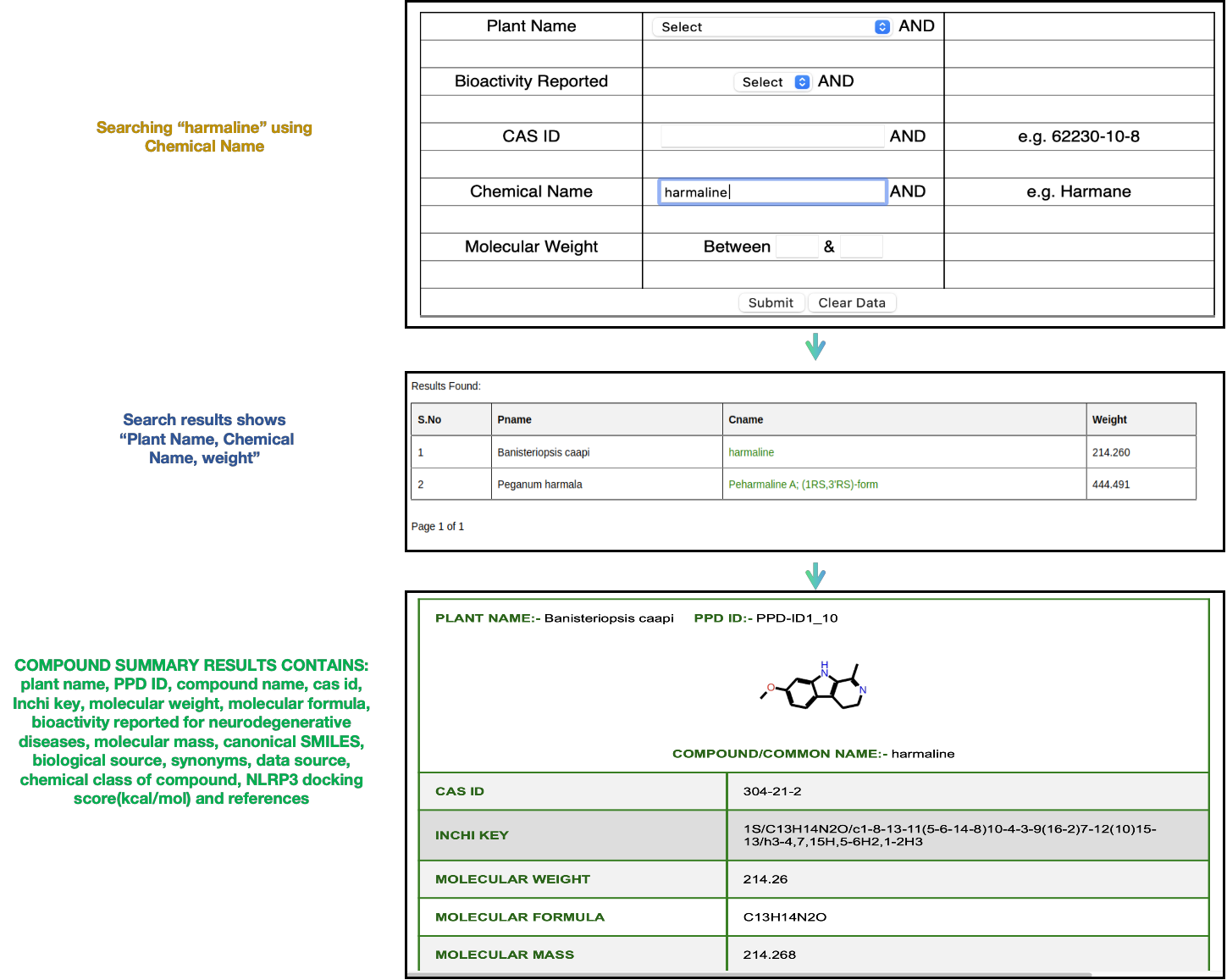

Supplement: Supplementary file 1 [file DataSheet1.docx]
